# A simple technique to avoid suturing in the drain during total knee arthroplasty

**DOI:** 10.1308/003588412X13373405387050e

**Published:** 2012-10

**Authors:** D Williams, K Sri-Ram

**Affiliations:** Royal National Orthopaedic Hospital NHS Trust,UK

## BACKGROUND

Closure of the arthrotomy following total knee arthroplasty is not without risk. Most orthopaedic surgeons are aware of cases when a drain has been caught by a suture during closure so that it cannot be removed, resulting in return to theatre, an intra-articular foreign body or infection.

## TECHNIQUE

**Figure 1:**
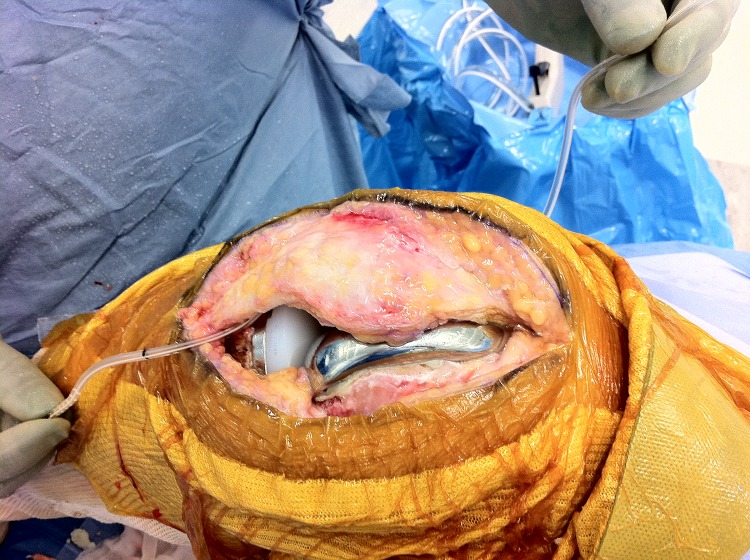
Clinical photograph showing the drain passing through the soft tissues lateral to the proximal end of the wound, leaving the distal end long

**Figure 2:**
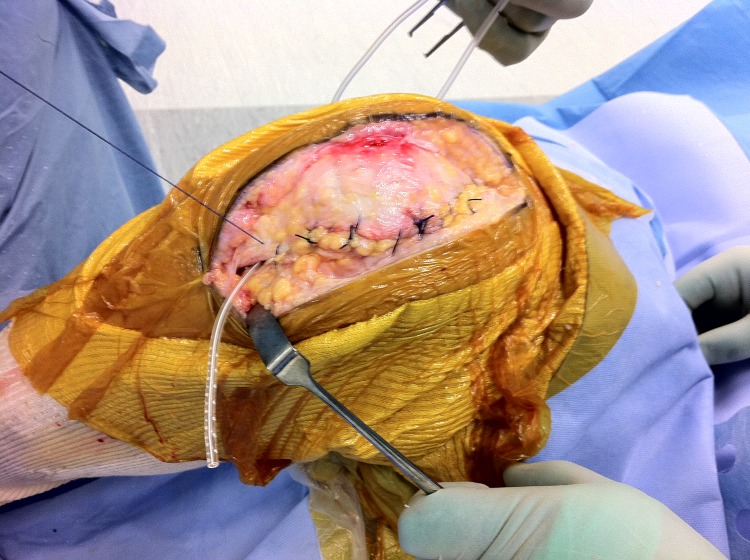
Clinical photograph demonstrating the long distal drain still protruding after closure of the arthrotomy

Before closing the arthrotomy, pass the drain through the soft tissues lateral to the proximal end of the wound, leaving the distal end long (Fig 1). Close the arthrotomy from proximal to distal, leaving the distal end protruding (Fig 2). As you reach the end of the wound where the drain exits the arthrotomy, withdraw the drain to length. As the drain withdraws easily, you can be sure that it has not been sutured into the wound. If it cannot be removed at this stage, the arthrotomy is easily explored.

## DISCUSSION

This simple technique reduces the chances and problems associated with a drain that cannot be removed after total knee arthroplasty. Although we acknowledge that this technique may not be novel, we would like to draw attention to its usefulness and feel it can be widely used in other procedures that have difficult closures.

